# 5-Azacytidine Improves the Osteogenic Differentiation Potential of Aged Human Adipose-Derived Mesenchymal Stem Cells by DNA Demethylation

**DOI:** 10.1371/journal.pone.0090846

**Published:** 2014-03-06

**Authors:** Xueying Yan, Sabrina Ehnert, Mihaela Culmes, Anastasia Bachmann, Claudine Seeliger, Lilianna Schyschka, Zhiyong Wang, Afshin Rahmanian-Schwarz, Ulrich Stöckle, Paul A. De Sousa, Jaroslav Pelisek, Andreas K. Nussler

**Affiliations:** 1 Siegfried Weller Institute for Trauma Research, BG Trauma Center, Eberhard Karls University Tübingen, Tübingen, Germany; 2 Clinic of Vascular and Endovascular Surgery, Klinikum rechts der Isar, Technische Universität München, Munich, Germany; 3 Department of Trauma Surgery, Klinikum rechts der Isar, Technische Universität München, Munich, Germany; 4 Clinic for Hand-, Plastic-, Reconstructive- and Vascular Surgery, BG Trauma Center, Eberhard Karls University Tübingen, Tübingen, Germany; 5 Center for Regenerative Medicine, the University of Edinburgh, Edinburgh, United Kingdom; Georgia Regents University, United States of America

## Abstract

The therapeutic value of adipose-derived mesenchymal stem cells (Ad-MSCs) for bone regeneration is critically discussed. A possible reason for reduced osteogenic potential may be an age-related deterioration of the Ad-MSCs. In long term *in vitro* culture, epigenomic changes in DNA methylation are known to cause gene silencing, affecting stem cell growth as well as the differentiation potential. In this study, we observed an age-related decline in proliferation of primary human Ad-MSCs. Decreased *Nanog*, *Oct4* and *Lin28A* and increased *Sox2* gene-expression was accompanied by an impaired osteogenic differentiation potential of Ad-MSCs isolated from old donors (>60 a) as compared to Ad-MSCs isolated from younger donors (<45 a). 5-hydroxymethylcytosine (5 hmC) and 5-methylcytonsine (5 mC) distribution as well as *TET* gene expression were evaluated to assess the evidence of active DNA demethylation. We observed a decrease of 5 hmC in Ad-MSCs from older donors. Incubation of these cells with 5-Azacytidine induced proliferation and improved the osteogenic differentiation potential in these cells. The increase in AP activity and matrix mineralization was associated with an increased presence of 5 hmC as well as with an increased *TET2* and *TET3* gene expression. Our data show, for the first time, a decrease of DNA hydroxymethylation in Ad-MSCs which correlates with donor-age and that treatment with 5-Azacytidine provides an approach which could be used to rejuvenate Ad-MSCs from aged donors.

## Introduction

Extensive bone loss after trauma or diseases often results in delayed or impaired bone healing [1]. Deteriorated bone regeneration and repair is observed with increasing age of patients [1], [2]. The use of mesenchymal stem cells (MSCs) in tissue engineering has great potential for a novel approach in acute bone and cartilage repair, e.g. some pioneer clinical study have already entered the phase III stage [3].

MSCs are one kind of adult stem cells. In the mechanism of osteogenesis, MSCs are considered to be one type of progenitor cells, which are able to proliferate and later on differentiate into osteogenic cells [4]. Hence, the process of bone regeneration requires the recruitment, expansion and differentiation of MSCs [5]. *In vitro* experiments demonstrated an active self-renewal capacity and multi-lineage differentiation potential of MSCs [6], [7]. MSCs can be isolated from various tissues; most frequently from adipose tissue and bone marrow. Due to their easy access, low immune rejection as well as a low risk of tumorigenesis [8], [9], MSCs derived from adipose tissue (Ad-MSCs) could be an ideal source for patient-specific cell therapy. However, the osteogenic differentiation potential of these Ad-MSCs has been critically discussed. Furthermore, it has been reported many times that adult stem cells, including MSCs, suffer from a decline in stem cell function with increasing age during long term culture of the cells [10], [11]. The decline observed in the self-renewal capacity, resulted in an incomplete differentiation into the committed cell lineage [12].

Epigenetic modification of the genome is considered to be one of the most important regulatory pathways affecting stem cell aging. These dynamic changes mainly appear in DNA methylation and/or chromatin remodeling [13]. Although selected histone-deacetylase inhibitors could improve osteogenic function in differentiated MSCs, they are not suitable for use because of their negative effects on cell proliferation due to DNA damage and cell-cycle inhibition [14]. Efforts have been made to investigate a milder epigenetic modification approach. It has been demonstrated that DNA demethylation can be induced by 5-Azacytidine, a DNA methyltransferase (DNMT) inhibitor [15]. In our hands 5-Azacytidine treatment improved the hepatic differentiation capacity of Ad-MSCs, in correlation to the DNA demethylation [16]. Just recently an active DNA-demethylation mechanism was described in embryonic stem cells. In these cells induction of *TET* gene expression promoted the conversion of nuclear 5-methylcytosine (5 mC) to 5-hydroxymethylcytosine (5 hmC) and thus DNA demethylation, in order to maintain the self-renewal capacity and the pluripotency state [17].

Thus, aim of the present study was to investigate donor-age-related changes in the self-renewal of Ad-MSCs as well as their osteogenic differentiation potential and which role DNA methylation plays in in this process. Furthermore, we want to investigate if these donor-age-related changes can be reversed by epigenetic modifications of the DNA. The stem cell capacity will be assessed by measuring the expression levels of transcription factors characteristic for embryonic and induced pluripotent stem cells, namely, *Sox2*, *Lin28A*, *Oct4* and *Nanog*. Cell proliferation will be determined by the amount of cells stained positive for Ki67. The osteogenic differentiation potential will be will be defined by measuring the AP-Activity and matrix mineralization after differentiation, as well as by measuring the expression levels of the osteogenic marker gene *osteocalcin* and the osteogenic transcription factors *Runx2* (early) and *osterix* (late) [18].

## Materials and Methods

Cell culture plastics, collagenase II, phosphate buffered saline (PBS), fetal calf serum (FCS), DMEM medium and cell culture supplements were purchased from PAA Laboratories GmbH (Pasching, Austria). GeneJET RNA Purification Kit, DNase I (RNase-free) and First Strand cDNA Synthesis Kit were purchased from Fermantas (Ontario, Canada). Oct-4A (C30A3) Rabbit mAB, Sox2 (D6D9) Rabbit mAB, Nanog (D73G4) Rabbit mAB, Lin28A (D84C11) Rabbit mAB and the corresponding secondary Anti-rabbit IgG, HRP linked Antibody were purchased from Cell Signaling (Beverly, USA). Ki67 (M-19) Goat pAB was purchased from Santa Cruz Biotechnology Inc. (Heidelberg, Germany). Chemicals as well as the Anti-TET2, Anti-TET3 and Anti-GAPDH Rabbit pAB were purchased from Sigma (Munich, Germany).

### Isolation, Expansion and Characterization of Ad-MSCs

Ad-MSCs were isolated from adipose tissue obtained from surgery with the written consent of the patients. This study was specifically approved by the ethical committee of the Universitätsklinikum Tübingen (Reference: 385/2012 B02) who works in accordance with national regulations and the ICH-GCP guidelines. The study was performed according to the declaration of Helsinki in its newest version. For this study adipose tissue from 23 donors was collected and isolated. Adipose tissue was minced and washed extensively with PBS before digestion with collagenase II (0.075% in PBS) at 37°C for 30 to 60 min. The digestion was stopped with culture medium (DMEM, 10% FCS, 100 U/ml penicillin, 100 µg/ml streptomycin). After centrifugation (600 g, 10 min) the cell pellet was collected, re-suspended and filtered through a 70 µM cell strainer. The cells were seeded in flasks which culture medium. To remove non-attached cells the Ad-MSCs were washed once with PBS 24 hours after isolation. Ad-MSCs were expanded in culture medium until passage 3 in order to minimize the number of contaminating cells in the culture. Purity of the Ad-MSCs was determined by flow cytometry as recently published by our group (CD90^+^, CD105^+^, CD14^−^, CD45^−^) [16].

### Pre-treatment of Ad-MSCs with 5-Azacytidine

Ad-MSCs from young (<45 a) and old (≥60 a) donors were seeded at a cell density of 10,000 cells/cm^2^. After cell attachment the medium was changed to freshly made culture medium containing 5-Azacytidine (5 µM and 20 µM). After 24 h the 5-Azacytidine containing culture medium was refreshed for another 24 h (total pre-treatment: 48 h).

### Osteogenic Differentiation of Ad-MSCs

For osteogenic differentiation Ad-MSCs from young and old donors were incubated with osteogenic differentiation medium (DMEM, 0.5% FCS, 100 U/ml penicillin, 100 µg/ml streptomycin, 100 µM L-Ascorbic Acid-2-phosphate, 10 mM β-glycerol phosphate, 25 mM HEPES, 1.5 mM CaCl_2_, 5 µM cholecalciferol) for 14 days. The differentiation medium was changed every 3–4 days.

### Alkaline Phosphatase (AP) Activity

In order to determine the AP activity, the differentiated cells were incubated with substrate solution (2 mg/ml 4-Nitrophenyl phosphate disodium salt hexahydrate, 50 mM glycine, 1 mM MgCl_2_, 100 mM Tris, pH = 10.5) for 120 min. The subsequent 4-Nitrophenol production in the supernatant was measured photometrically at λ = 405 nm with a FLUOstar Omega Plate reader (BMG Labtech, Offenburg, Germany), and normalized to relative cell numbers measured by Sulforhodamine B Staining as described before [19], [20].

### Matrix Mineralization

Matrix mineralization was assessed by Alizarin red and von Kossa staining. For both stainings cells were fixed with 99% ethanol for 1 h at −20°C. For alizarin red staining cells were incubated with 0.5% Alizarin red S solution (PH = 4.0) for 20 min, then washed extensively with tap water to remove unbound stain. For von Kossa staining cells were incubated with 3% AgNO_3_ solution for 30 min, then washed extensively with tap water, exposed to 5% sodium carbonate (in 10% formaldehyde) solution for 2 min, followed by an incubation in 5% sodium-thiosulfate solution for 5 min in order to develop color. For both staining methods the plates were first scanned to obtain an overview image, than images with the light microscope were taken. Alizarin red staining was quantified photometrically (λ = 562 nm) with a FLUOstar Omega Plate reader, after resolving the stain with 10% cetylpyridinium chloride solution. The von Kossa staining was quantified densitometrically (Pixel intensity) with the Image J. 1.45 s software (National Institutes of Health, USA).

### Immunofluorescence Staining

Ad-MSCs were plated onto cover slips and treated according to the experimental setup. Cells were fixed with 4% paraformaldehyde solution for 15 min at RT and then washed with PBS. For permeabilization, cells were incubated with 0.5% Triton-X-100 PBS solution for 30 min at RT. To denature the DNA cells were incubated with 4 M HCl for 15 min at RT. After washing with PBS unspecific binding sites were blocked with blocking buffer (10% FCS, 0.1% Tween-20 in PBS) for 1 h at RT. Then cells were incubated with primary antibody solution anti-5 hmC rabbit polyclonal IgG (Active Motif, CA, USA), anti-5 mC mouse monoclonal IgG, (Active Motif, CA, USA) or anti-Ki67 goat polyclonal IgG (Santa Cruz Biotechnology, CA, USA) diluted 1∶100 in PBS solution containing 1% FCS and 0.1% Tween-20 for 2 h at RT. After washing three times with PBS cells were incubated with secondary antibody solution (ALEXA-fluor antibodies (Invitrogen, NY, USA) diluted 1∶400 in PBS solution containing 1% FCS, 0.1% Tween-20 for 1 h at RT. Nuclei were counterstained by incubation with Hoechst 33342 solution (2 µg/ml in PBS) for 20 min at RT. After a final washing step with PBS, the stained cells were mounted with mounting medium (Fluoromount G, Southern Biotech, NJ, USA). Images of the staining were taken with an EVOS fluorescence microscope (AMG, USA) under standardized condition, processed and analyzed with Image J 1.45 s software [21].

### Quantification of Gene Expression

Total RNA from Ad-MSCs was extracted with GeneJET RNA Purification Kit. 2–3 µg of the total RNA was digested with DNase I in order to remove remaining genomic DNA. cDNA was synthesized using the First Strand cDNA Synthesis Kit. Gene expression changes were analyzed either by conventional RT-PCR or by qRT-PCR depending on the amount of target mRNA present in the sample. PCR protocols were optimized for each gene separately to be in a quantifiable (linear) range in our samples.

For quantitative real time PCR (qRT-PCR) a standardized amount of template cDNA was tested for the expression level of each target gene (primer sequences are listed in Table 1) using SYBR Green qPCR (Finnzym, Vantaa, Finnland) and the Step One Plus Real-Time PCR System (Applied Biosystems, CA, USA). *GAPDH* was used as endogenous control. Relative changes in gene expression were calculated using the ΔΔC_T_ Method with either untreated young cells (changes with age) or untreated age matched cells (changes du to 5-Azacytidine treatment) as reference.

**Table 1:** Sequence of primers used in qRT-PCR.

	gene bank ID	Forward primer (5′->3′)	reverse primer (5′->3′)	product length	T_a_	# of cycles
*Runx2*	NM_001015051.3	TGCCTAGGCGCATTTCAGGTGC	GGTGGTGGTGCATGGCGGAA	359 bp	58°C	30*
*Osterix*	NM_152860	CCCAGGCAACACTCCTACTC	GGCTGGATTAAGGGGAGCAAA	175 bp	62°C	30*
*Osteocalcin*	NM_199173.3	CCAGCGGTGCAGAGTCCAGC	GACACCCTAGACCGGGCCGT	236 bp	56°C	25*
*TET1*	NM_030625.2	GTAAATGGCCCCAAGTCAGA	CAGCTTCTGGGACATTAGCA	211 bp	59°C	40 (qPCR)
*TET2*	NM_001127208.2	GAGACGCTGAGGAAATACGG	TGGTGCCATAAGAGTGGACA	258 bp	59°C	40 (qPCR)
*TET3*	NM_144993.1	CAGAACGCTGTGATCGTCAT	AACTTGCGAGGTGTCTTGCT	263 bp	59°C	40 (qPCR)
*NANOG*	NM_024865.2	AACTGGCCGAAGAATAGCAA	ACTGGATGTTCTGGGTCTGG	175 bp	59°C	40 (qPCR)
*Oct4*	NM_002701.4	AGTGAGAGGCAACCTGGAGA	GCCTCAAAATCCTCTCGTTG	180 bp	59°C	40 (qPCR)
*Lin28A*	NM_024674.4	CCGAACCCCATGCGCACGTT	TTTGCAGGTGGCTGCGCCAAG	137 bp	59°C	40 (qPCR)
*Sox2*	NM_003106.3	CATGCACCGCTACGACG	CGGACTTGACCACCGAAC	152 bp	62°C	40 (qPCR)
*GAPDH*	NM_002046.4	TGCACCACCAACTGCTTAGC	GGCATGGACTGTGGTCATGAG	87 bp	59°C	40 (qPCR)
*Runx2*	NM_001015051.3	TGCCTAGGCGCATTTCAGGTGC	GGTGGTGGTGCATGGCGGAA	359 bp	58°C	30*

*PCR conditions were optimized with varying template concentrations to be in the quantifiable/linear range of the PCR.

For semi-quantitative (conventional) RT-PCR a standardized amount of template cDNA was tested for the expression level of each target gene (primer sequences are listed in Table 1) using KAPA Fast ready mix (Peqlab, Erlangen, Germany). Products, resolved by gel electrophoresis in a 2% (w/v) agarose gel, were visualized with ethidiumbromide. Densitometric analysis of signals was performed using ImageJ software (NIH, Bethesda, USA). Pixel Intensities of the PCR signal were normalized to *GAPDH* using a normalization factor (multiple of the average GAPDH signal). Relative changes in gene expression were calculated with either untreated young cells (changes with age) or untreated age matched cells (changes du to 5-Azacytidine treatment) as reference.

### Western Blot

Cells were lyzed in freshly prepared ice-cold RIPA buffer (50 mM TRIS, 250 mM NaCl, 2% Nonidet-P40, 2.5 mM EDTA, 0.1% SDS, 0.5% DOC, protease and phosphatase inhibitors, pH = 7.2). Protein concentration was determined by micro-Lowry [22]. 50 µg total protein were separated by SDS-PAGE and transferred to nitrocellulose membranes (Roth, Karlsruhe, Germany). Unspecific binding sites were blocked with 5% BSA in TBST solution (25 mM Tris, 137 mM NaCl, 2.7 mM KCl, 0.05% Tween-20, pH = 7.4) for 1 h at RT. After overnight incubation with primary antibodies diluted 1∶1,000 in TBST at 4°C, membranes were incubated with the corresponding HRP-labeled secondary antibodies (1∶10,000 in TBST) for 2 h at RT. For signal development membranes were incubated for 1 min with ECL substrate solution (1.25 mM luminol, 0.2 mM p-coumaric acid, 0.03% H_2_O_2_ in 100 mM TRIS, pH = 8.5). Chemiluminescent signals were detected with a CCD camera (INTAS, Göttingen, Germany). GAPDH was used as loading control. Densitometric analysis of signals was performed using ImageJ software (NIH, Bethesda, USA).

### Statistical Analysis

Results are expressed as box blot (Whisker’s: min to max) or bar chart (mean ± SEM) of at least 3 independent experiments (biological replicates, N≥3) measured as triplicates or more (technical replicates, n≥3). The precise of replicates for each experiment is given in the figure legends. Individual data sets were compared by Student’s t test or one way ANOVA analysis (Prism 5.04, GraphPad Software, CA, USA), with consideration of the sample distribution. *p*<0.05 was set as the minimum level of significance.

## Results

### Ad-MSCs from Elderly Donors showed Impaired Proliferation and Osteogenic Differentiation Capacity

**Figure 1:**
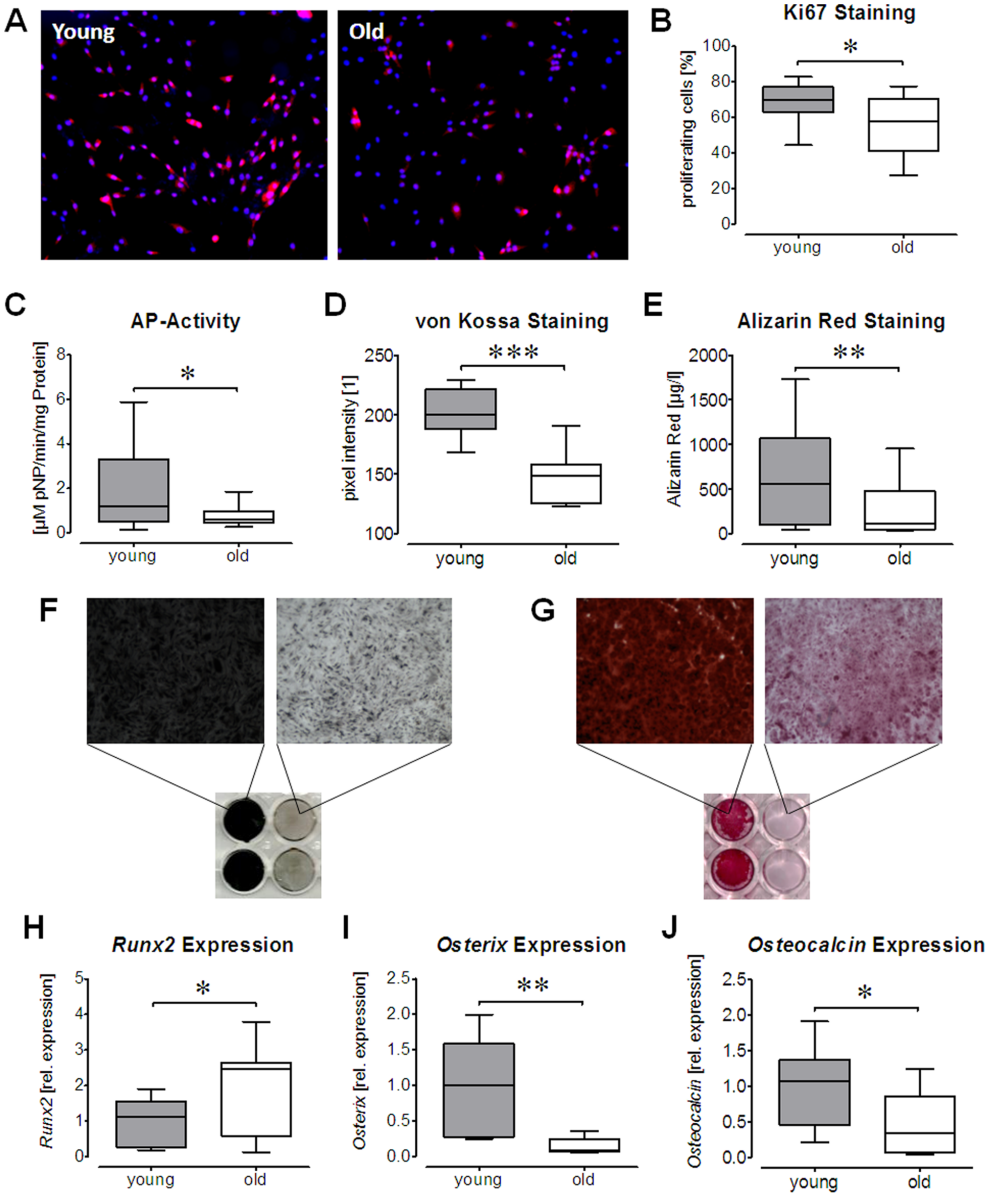
Declined Proliferation and Osteogenic Differentiation in Ad-MSCs from old donors: Ki67 staining was performed as proliferation assay. (A) Representative image of a merged immunofluorescent staining for Ki67 (red) and nuclei (blue) from young and old Ad-MSCs. (B) Quantification of Ki67-positive Ad-MSCs (N = 3, n = 5/age group) points to a reduced proliferation in aged Ad-MSCs. After 14 days of osteogenic differentiation of young and old Ad-MSCs osteoblasts function was assessed by measuring (C) AP activity (each group: N = 7, n = 3), (D–G) matrix mineralization and (H–J) gene expression changes. Matrix mineralization was determined by (D & F) von Kossa (each group: N = 5, n = 3) and (E & G) Alizarin red staining (each group: N = 6, n = 3). mRNA levels of the osteogenic transcription factors (H) *Runx2* and (I) *Osterix*, as well as the osteogenic marker (J) *Osteocalcin* were determined by semi-quantitative RT-PCR (each group: N = 5, n = 3). *GAPDH* was used for normalization. *p<0.05, **p<0.01, ***p<0.001 (student’s t-test).

In order to assess the proliferation capacity of the cells a Ki67 staining was performed. Expression of Ki67 was significantly lower in Ad-MSCs isolated from aged donors as compared to Ad-MSCs isolated from younger donors (figure 1A & B), representing an impaired proliferation capacity of Ad-MSCs from elderly donors. In order to evaluate the differentiation potential, Ad-MSCs were differentiated for 14 days in the presence of osteogenic differentiation medium. As osteogenic markers alkaline phosphatase (AP) activity, matrix mineralization and relative gene expression of *Runx2*, *osterix* and *osteocalcin* were determined after the differentiation process. In comparison to Ad-MSCs from young donors, Ad-MSCs from old donors exhibited a significantly lower AP activity (figure 1C) and a significant weaker matrix mineralization, as determined by von Kossa (figure 1D & F) and Alizarin red staining (figure 1E & G). Increased expression of the early osteogenic transcription factor *Runx2* and reduced expression of the late osteogenic transcription factor *osterix* suggest a delay in osteogenic differentiation in Ad-MSCs from aged donors. This is supported by reduced expression of *osteocalcin*, the most abundant non-collagenous protein in bone (figure 1H–J).

### Gene Expression of Pluripotency Markers Demonstrated Age-related Differences in Ad-MSCs

**Figure 2:**
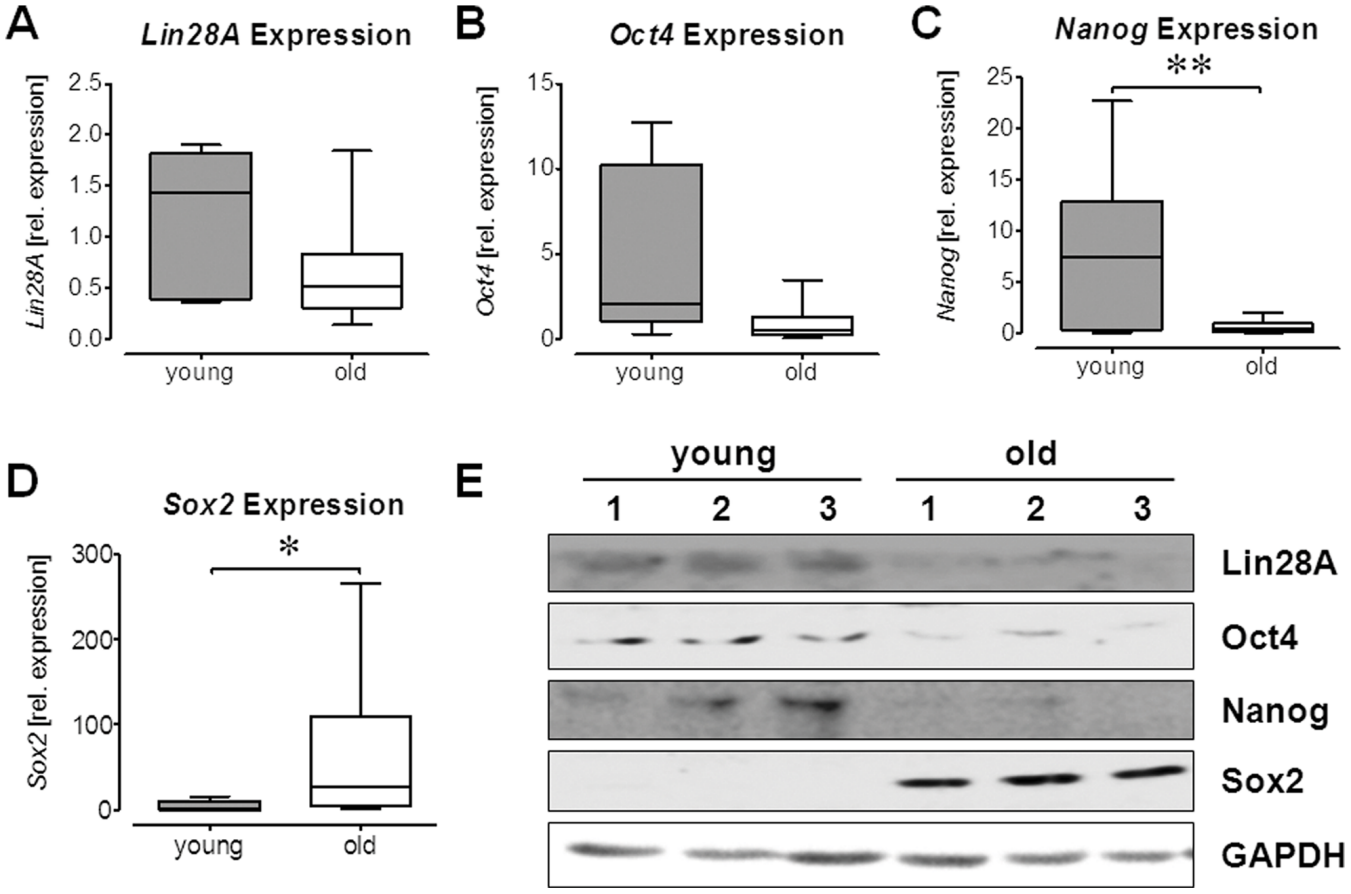
Altered expression of pluripotency genes in Ad-MSCs from old donors. Basal expression levels of pluripotency genes were determined in Ad-MSCs from young and old donors (each group: N = 6, n = 3). mRNA levels of (A) *Lin28A* (2 donors from the young Ad-MSCs had to be excluded from the data set, due to artefacts in the melting curve), (B) *Oct4*, (C) *Nanog* and (D) *Sox2* were determined by qRT-PCR. (E) Expression changes of *Lin28A*, *Oct4*, *Nanog* and *Sox2* were confirmed on the protein level by Western blot analysis (N = 3/group). For both techniques *GAPDH* was used for normalization. *p<0.05, **p<0.01 (student’s t-test).

The differentiation potential was evaluated by relative gene expression levels of the pluripotency markers *Nanog*, *Oct4*, *Lin28A* and *Sox2* by qRT-PCR. GAPDH was used as endogenous control. Expression of *Lin28A* was significantly lower in Ad-MSCs from elderly donors (figure 2A). Similarly, expression of *Oct4* was reduced in Ad-MSCs from aged donors (figure 2B). Furthermore, expression of *Nanog* was significantly lower in Ad-MScs from elderly donors (figure 2C). Conversely, expression levels of *Sox2* were significantly elevated in Ad-MSCs from aged donors (figure 2D). The observed expression changes were confirmed on protein level by Western blot (figure 2E).

### TET Gene Expression and 5 mC/5 hmC Distribution in Ad-MSCs from Young and Old Donors

**Figure 3:**
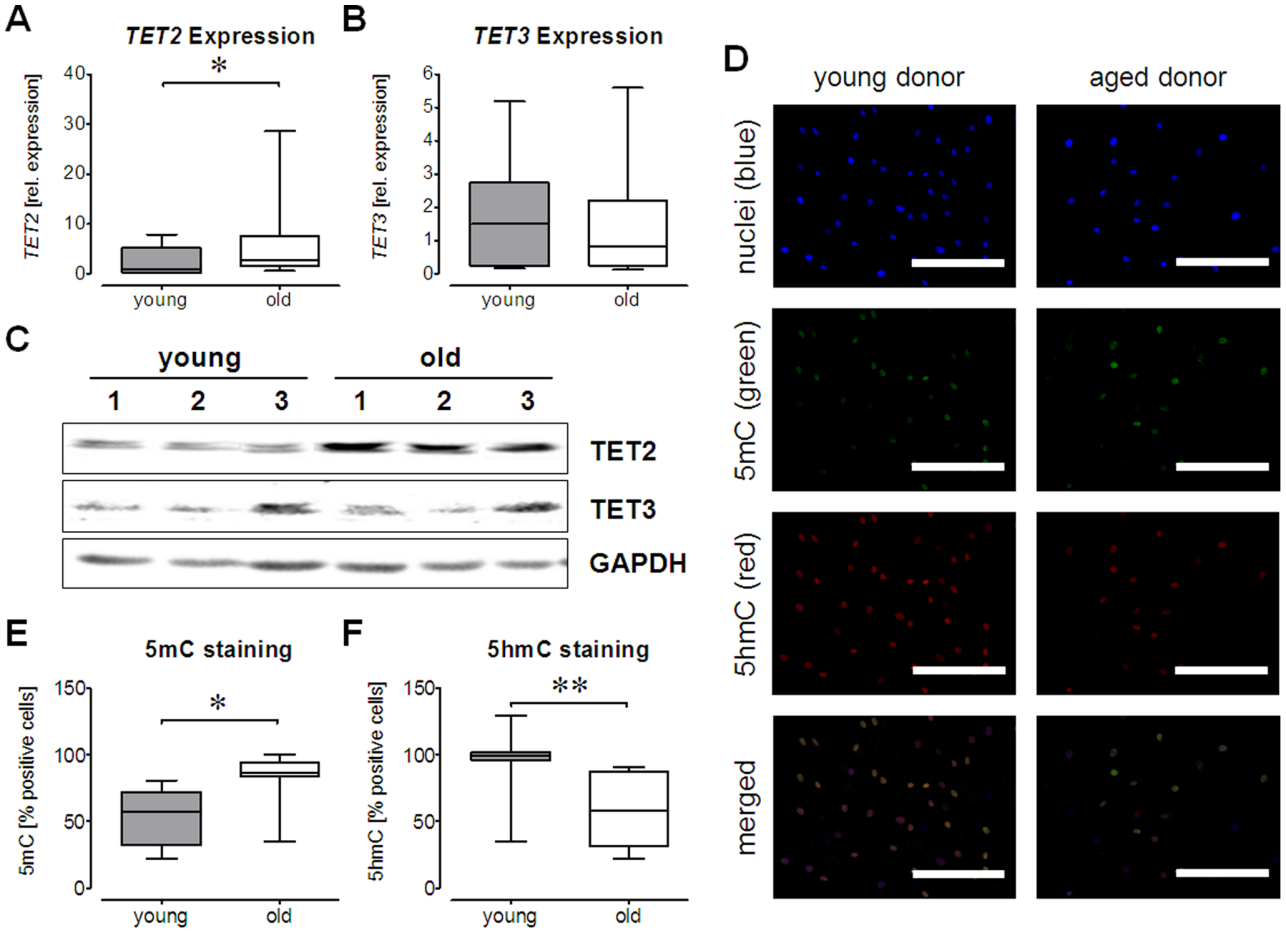
Distribution of 5-hydroxymethylcytosine (5 hmC) and 5-methylcytosine (5 mC) in Ad-MSCs. Basal mRNA levels of (A) *TET2* and (B) *TET3* were determined by qRT-PCR in Ad-MSCs from young and old donors (each group: N = 6, n = 3). (C) Expression changes of *TET2* and *TET3* were confirmed on the protein level by Western blot analysis (N = 3/group). For both techniques *GAPDH* was used for normalization. (D) Young and old Ad-MSCs (each group: N = 3, n = 5) were stained for 5-hydroxymethylcytosine (5 hmC/red) and 5-methylcytosine (5 mC/green). Cell nuclei were counterstained with Hoechst 33342 (blue). (E&F) For quantification 5 hmC or 5 mC positive nuclei were counted. *p<0.05, **p<0.01 (student’s t-test).

Members of the *TET* family (*TET*1-3) are reported to actively regulate DNA methylation. Therefore, we determined relative gene expression levels of *TET1*, *TET2* and *TET3* by qRT-PCR. GAPDH was used as endogenous control. Expression of *TET1* was near/below the detection limit in Ad-MSCs from young and elderly donors. Therefore, no significant difference could be detected (data not shown). Expression levels of *TET2* were significantly elevated in Ad-MSCs from aged donors (figure 3A). *TET3* expression levels were not changed between the two age groups (figure 3B). The observed expression changes were confirmed on protein level by Western blot (figure 3C). Genomic distribution of 5-hydroxymethylcytosine (5 hmC) and 5-methylcytosine (5 mC) in Ad-MSCs from young and old donors was evaluated by immunofluorescence staining (5 hmC: red, 5 mC: green) in order to assess the DNA methylation status. Nuclei were counterstained with Hoechst 33342 (blue) (figure 3D). The 5 mC-positive fraction was significantly higher in the Ad-MSCs from old donors than in the Ad-MSCs from young donors (figure 3E). The 5 hmC-positive fraction was significantly lower in the Ad-MSCs from old donors than in the Ad-MSCs from young donors (figure 3F).

### 5-Azacytidine Treatment Induced Expression of TET2 and TET3 while Increasing Nuclear 5 hmC Levels

**Figure 4:**
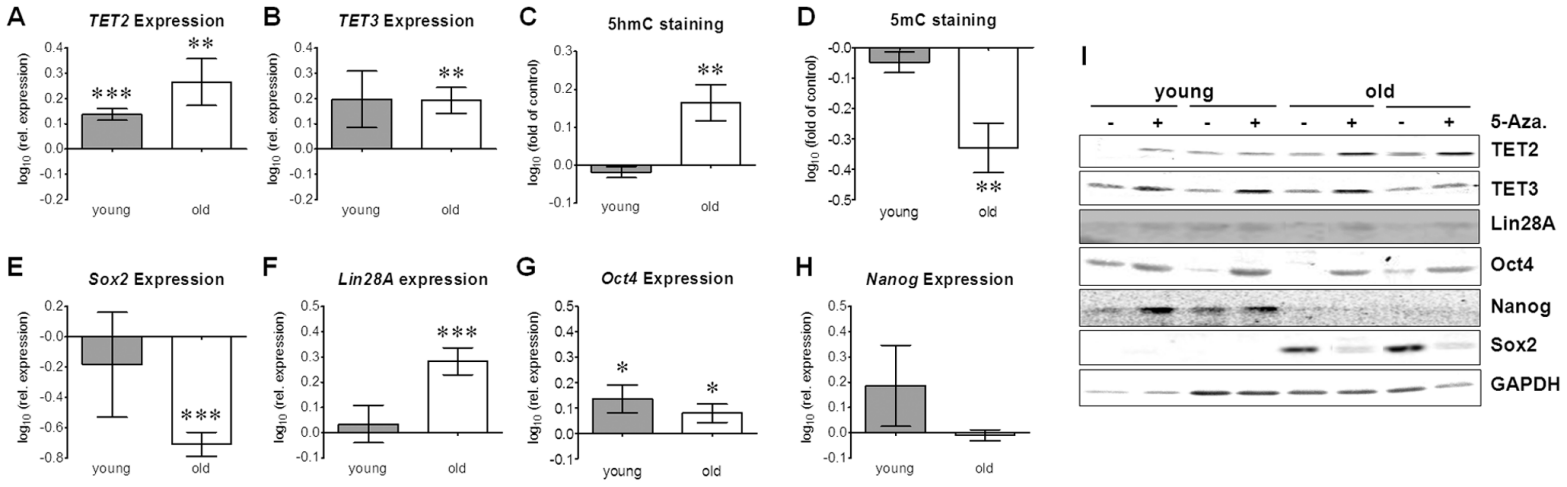
5-Azacytidine treatment increased *TET2* and *TET3* gene expression. Young and old Ad-MSCs were treated with 5 µM 5-Azacytidine for 48 h. mRNA levels of (A) *TET2*, (B) *TET3*, (E) Sox2, (F) *Lin28A* (2 donors from the young Ad-MSCs had to be excluded from the data set, due to artefacts in the melting curve), (G) *Oct4* and (H) *Nanog* were determined by qRT-PCR (N = 6, n = 3/age group). (I) Expression changes of *TET2, TET3, Lin28A*, *Oct4*, *Nanog* and *Sox2* were confirmed on the protein level by Western blot analysis (Representative figure for N = 2/group). *GAPDH* was used for normalization. Data are presented as relative expression changes (log_10_) of 5-Azacytidine treated Ad-MSCs compared to the corresponding untreated Ad-MSCs. Distribution of 5-hydroxymethylcytosine (5 hmC) and 5-methylcytosine (5 mC) in the 5-Azacytidine treated and untreated Ad-MSCs was determined by immunofluorescent staining (N = 3, n = 5/age group). Cell nuclei were counterstained with Hoechst 33342. For quantification (D) 5 hmC and (E) 5 mC positive nuclei were counted. Data are represented as log_10_ (fold of control). *p<0.05, **p<0.01, ***p<0.001 as compared to untreated cells (student’s t-test).

5-Azacytidine is a well characterized DNA-methyltransferase inhibitor. In order to evaluate the effect of 5-Azacytidine on the DNA methylation status, relative gene expression levels of *TET1*, *TET2* and *TET3* were determined by qRT-PCR in Ad-MSCs from young and old donors treated for 48 h with 5 µM 5-Azacytidine. *GAPDH* was used as endogenous control. After 5-Azacytidine treatment *TET1* expression was still near the detection limit in Ad-MSCs from both young and old donors, where no significant difference could be detected (data not shown). In both age groups, *TET2* expression levels were significantly increased by 5-Azacytidine treatment (figure 4A). Similarly, *TET3* expression levels were increased by 5-Azacytidine treatment in both age groups, with more pronounced changes in Ad-MScs from aged donors (figure 4B). Expression changes of TET2 and TET3 due to 5-Azacytidine treatment were confirmed by Western blot on the protein level (data not shown). Genomic distribution of 5 hmC and 5 mC, in Ad-MSCs from old donors, was strongly affected by 5-Azacytidine treatment, as determined by immunofluorescence staining. The 5 hmC-positive fraction was significantly increased in Ad-MSCs from old donors after treatment with 5-Azacytidine (figure 4C). This resulted in a significant decrease in the 5 mC-positive fraction in Ad-MSCs from old donors after treatment with 5-Azacytidine (figure 4D). Consequently relative expression levels of the pluripotency markers *Nanog*, *Oct4*, *Lin28A* and *Sox2* were changed by 5-Azacytidine treatment, as determined by qRT-PCR. Expression levels of *Sox2* were significantly decreased by 5-Azacytidine treatment only in Ad-MSCs from aged donors (figure 4E). Expression of *Lin28A* was significantly induced by 5-Azacytidine treatment in Ad-MSCs from elderly donors (figure 4F). Expression of *Oct4* was significantly induced by 5-Azacytidine treatment in Ad-MSCs from both age groups (figure 4G). In contrary to *Lin28A* expression of *Nanog* was induced by 5-Azacytidine treatment only in Ad-MScs from young donors (figure 4H). On the protein level, pluripotency markers showed a comparable regulation due to 5-Azacytidine treatment (figure 4I).

### 5-Azacytidine Pre-treatment Improved Proliferation and Osteogenic Differentiation of Ad-MSCs from Aged Donors

**Figure 5:**
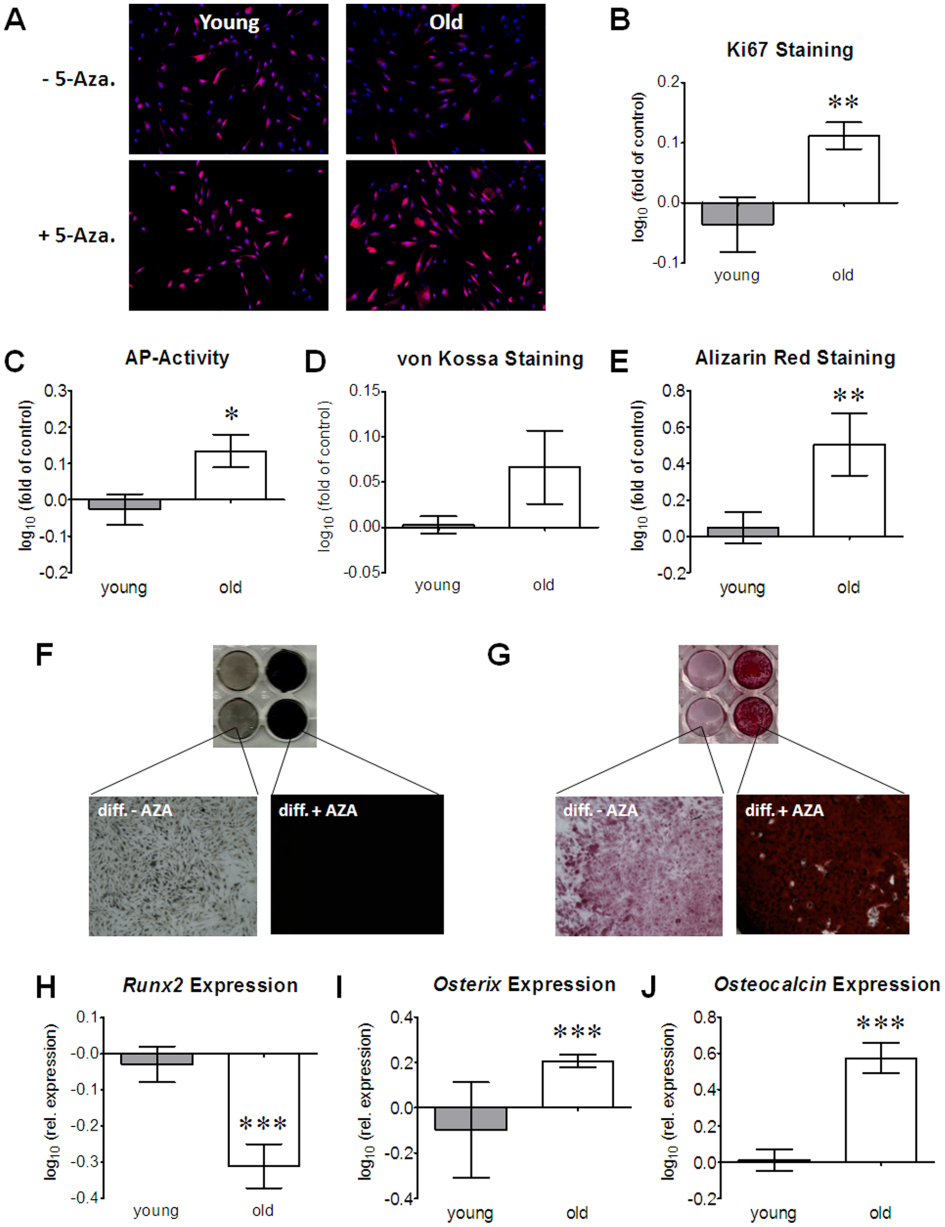
5-Azacytidine treatment improves osteogenic differentiation of Ad-MSCs from old donors. Ki67 staining was performed to assess the proliferation capacity of young and old Ad-MSCs after treatment with 5 µM 5-Azacytidine for 48 h. (A) Representative image of a merged immunofluorescent staining for Ki67 (red) and nuclei (blue). (B) Quantification of Ki67-positive Ad-MSCs (N = 3, n = 5/age group) points to an induced proliferation in aged Ad-MSCs after treatment with 5-Azacytidine. Young and old Ad-MSCs were pre-treated with 5 µM 5-Azacytidine for 48 h before osteogenic differentiation was induced. In the differentiated cells osteoblasts function was assessed by measuring (C) AP activity (N = 7, n = 3/age group), (D–G) matrix mineralization and (H–J) gene expression changes. Matrix mineralization was determined by (D & F) von Kossa (N = 4, n = 3/age group) and (E & G) Alizarin red staining (N = 6, n = 3/age group). mRNA levels of the osteogenic transcription factors (H) *Runx2* and (I) *Osterix*, as well as the osteogenic marker (J) *Osteocalcin* were determined by semi-quantitative RT-PCR (N = 5, n = 3/age group). GAPDH was used for normalization. All data are presents as relative changes (log_10_) of 5-Azacytidine pre-treated differentiated Ad-MSCs compared to the corresponding differentiated Ad-MSCs without pre-treatment.*p<0.05, **p<0.01, ***p<0.001 as compared to differentiated Ad-MSCs without pre-treatment (student’s t-test).

In the following experiments we wanted to analyze of the reduction in DNA methylation by 5-Azacytidine improves the proliferation and the osteogenic differentiation capacity of aged Ad-MSCs. Following the treatment of Ad-MSCs from old donors with 5 µM 5-Azacytidine for 48 h significantly more cells were Ki67-positive (1.3-fold, *p*<0.01), representing an improved proliferation. Conversely, no improvement of the proliferation was observed in Ad-MSCs from young donors following the treatment with 5 µM 5-Azacytidine for 48 h (figure 5A & B). After pre-treatment with 5 µM 5-Azacytidine for 48 h, Ad-MSCs from aged donors were differentiated for 14 days in the presence of osteogenic differentiation medium. As osteogenic markers alkaline phosphatase (AP) activity, matrix mineralization and relative gene expression of *Runx2*, *osterix* and *osteocalcin* were determined after the differentiation process. Pre-treatment with 5-Azacytidine improved AP activity (figure 5C) only in Ad-MSCs from elderly donors. Similarly, matrix mineralization was enhanced by 5-Azacytidine treatment only in Ad-MSCs from aged donors as determined by von Kossa (figure 5D & F) and Alizarin red staining (figure 5E & G). Expression levels of the early osteogenic transcription factor *Runx2* were significantly reduced by 5-Azacytidine treatment only in Ad-MSCs from aged donors (figure 5H). Conversely, expression levels of the late osteogenic markers *osterix* and *osteocalcin* were significantly induced by 5-Azacytidine treatment only in Ad-MSCs from elderly donors (figure 5I & J).

## Discussion

Stem cell functions include self-renewal and multipotency or even pluripotency. In particular, self-renewal of stem cells does not only include proliferation, but also the maintenance of their differentiation potential after cell division [23]. Our results demonstrate a decreased proliferation rate of Ad-MSCs from aged donors as well as an impaired osteogenic differentiation potential, as characterized by a declined AP activity, a weaker mineralization of extracellular matrix and reduced expression of late osteogenic marker genes *osterix* and *osteocalcin* in the differentiated cells from elderly donors, which was accompanied by a strong DNA methylation. Increased global DNA methylation status is a well-established phenomenon that has been observed in many aged adult tissue/cell types [24].

Our preliminary research has shown that 5-Azacytidine treatment significantly reduces the global level of DNA methylation [16]. In the past, the demethylation effect of 5-Azacytidine was described as a cause of direct or indirect inhibition of the DNA methyltranferases (DNMTs) [25]. This theory resembles mainly the passive DNA demethylation process. Recently, more active mechanisms of DNA demethylation have been uncovered [26]. *TET* proteins mediated conversion of 5 mC to 5 hmC was shown to play a crucial role in active demethylation of DNA [27]. Here, we have presented for the first time, that Ad-MSCs display reduced levels of 5 hmC during aging. With the conversion of 5 mC to 5 hmC by *TET* proteins [17], further modifications can occur in order to launch the active DNA demethylation, *i.e.* oxidation to 5-formylcytosine (5 fC) and 5-carbonylcytosine (5 caC). 5 caC eventually will be removed by a base excision pathway and thus will be replaced with unmethylated cytosine [26]. Thus, the finding of reduced 5 hmC levels in aged Ad-MSCs could account for a DNA hypermethylation pattern in these [16] and other adult cell types [24]. This is further supported by the finding that 5 hmC has been proven to act like a poor substrate for DNMT1 recognition [28]. In this way, it is also able to take part in the passive DNA demethylation by preventing factors that interact with methylcytosine [26].

Most interestingly, treatment of aged Ad-MSCs with the DNMT-inhibitor 5-Azacytidine not only reduced DNA methylation passively [16] but also actively, by inducing *TET* expression and nuclear 5 hmC. In contrast to embryonic stem cells, where only *TET1* plays a key role in DNA hydroxylation [17], [29], in aged Ad-MSCs *TET2* and *TET3* expression was significantly induced by 5-Azacytidine treatment. Controversially to the hypothesis that increased *TET* levels lead to an accumulation of 5 hmC, aged Ad-MSCs show slightly increased basal expression levels of *TET2.* However, this hypothesis is based on work with embryonic stem cells [17], [29], where *TET*1 is the master regulator. In our cells from both age groups, however, *TET1* expression is negligible as it is close to/below the detection limit. Furthermore, it is still unclear, whether expression and activity of DNA methyl-transferases is altered with aging as suggested by Casillas and co-workers [30].

The work of Koh *et al* reports that expression of *TET1* and *TET2* is strongly dependent on the expression of Oct4, while *TET3* expression negatively correlates with *Sox2* expression levels [31]. In Ad-MSCs from elderly donors expression of the pluripotency-associated genes *Nanog, Oct4* and *Lin28A* was strongly reduced, while *Sox2* expression was induced. After 5-Azacytidine *Oct4* and *Lin28A* expression significantly increased and *Sox2* expression was significantly decreased in aged Ad-MSCs. Considering the data from Koh *et al* this might explain the observed increase in expression of *TET2* and *TET3* after 5-Azacytidine treatment in Ad-MSCs from aged donors [31]. Why *TET2* expression was not affected by 5-Azacytidine treatment in Ad-MSCs from young donors might be explained by the interaction of the different pluripotency factors itself [32], as in this age group 5-Azacytidine treatment induced the expression of *Oct4* and *Nanog* but not *Lin28A*.

*Oct4* and *Nanog* are described as core transcription factors for the regulation and the maintenance of pluripotency in embryonic stem cells [33], [34]. It has been reported that *Lin28A* is able to promote cell proliferation in embryonic stem cells and breast tumor cells [35], [36]. Our data imply that the observed age-related loss of capacity for self-renewal and differentiation could be triggered by the reduced expression of these and other genes controlling stem cell renewal. This implication was supported by the work of Tsai *et al*, showing, that with increasing time in culture, *Nanog* and *Oct4* expression as well as osteogenic differentiation capacity decreases in MSCs [37]. *Lin28A* and *Oct4* may play a special role in this regulatory mechanism as the treatment with 5-Azacytidine, which strongly induced the expression of *Lin28A* and *Oct4*, was able to improve proliferation and osteogenic differentiation in Ad-MSCs from elderly donors. Basal expression levels of the pluripotency factor *Sox2* was very low in young Ad-MSCs, which is in line with other publications [38]. Interestingly, basal *Sox2* expression levels were significantly elevated in Ad-MSCs from elderly donors and decreased with the 5-Azacytidine treatment. The decline in *Sox2* expression after 5-Azacytidine treatment may be pivotal for the improved osteogenic differentiation, as there are reports showing that depletion of *Sox2* promotes osteogenic differentiation in a great variety of stem cells [39], [40], [41], [42].

In our aged Ad-MSCs 5-Azacytidine not only induced the cells proliferation but also accelerated their osteogenic differentiation. This is shown by a significant increase in AP activity and matrix mineralization. Supporting this hypothesis the early osteogenic transcription factor *Runx2* is reduced in favor for a faster induction of the late osteogenic marker genes *osterix* and *osteocalcin* [18].

## Conclusion

Summarizing, our data suggest that DNA methylation plays a crucial role in adult stem cell aging, as DNA methylation increases with donor age in Ad-MSCs. We could show that 5-Azacytidine, a small molecule inhibitor for DNMTs, not only passively but also actively promotes DNA demethylation in Ad-MSCs from aged donors. This balanced the expression levels of pluripotency genes and improved the osteogenic differentiation potential, thus rejuvenating aged Ad-MSCs.
